# Self-assembly and clustering of magnetic peapod-like rods with tunable directional interaction

**DOI:** 10.1371/journal.pone.0195552

**Published:** 2018-04-09

**Authors:** Jorge L. C. Domingos, François M. Peeters, W. P. Ferreira

**Affiliations:** 1 Departamento de Física, Universidade Federal do Ceará, Fortaleza, Ceará, Brasil; 2 Department of Physics, University of Antwerp, Groenenborgerlaan 171, B-2020 Antwerpen, Belgium; Universidade de Lisboa, PORTUGAL

## Abstract

Based on extensive Langevin Dynamics simulations we investigate the structural properties of a two-dimensional ensemble of magnetic rods with a peapod-like morphology, i.e, rods consisting of aligned single dipolar beads. Self-assembled configurations are studied for different directions of the dipole with respect to the rod axis. We found that with increasing misalignment of the dipole from the rod axis, the smaller the packing fraction at which the percolation transition is found. For the same density, the system exhibits different aggregation states for different misalignment. We also study the stability of the percolated structures with respect to temperature, which is found to be affected by the microstructure of the assembly of rods.

## Introduction

Many efforts are currently devoted to the search of new functionalized particles in order to satisfy the constant need for materials with different properties. Recent advances in particle synthesis has resulted in a rapid growth of this field of material science. Colloids with directional interactions are promising candidates [1–3]. By tuning the direction, the self-assembling process can be controlled leading to the emergence of specific structures. A paradigm example are dipolar colloids whose interactions are governed by permanent or field-induced, magnetic or electric dipole moments, as well as particles with more complex multipolar interactions.

Nanoparticles (NP) with a magnetic mono-domain (MN), are a typical example with a wide range of applications, including magnetic fluids [4], biomedicine [5], Magnetic Resonance Imaging (MRI) [6], data storage [7], among others. Currently growing attention is being paid to magnetic particles with anisotropic shape, due to their more complex properties when compared to those with spherical shape, as for example magnetic birefringence [8] and thermal conductivity [9]. Beyond anisotropy in the shape of the particles, the same attention was also addressed to cases where the anisotropy is in the location of the dipole with respect to the center of symmetry of the particle. In recent theoretical works the structure of fluids containing spherical particles with embedded off-centered magnetic dipoles [10, 11] was investigated.

Beyond the nature of the interaction itself, the morphology of the NP can be used as a controlling parameter to functionalize the MN through the interaction direction, i.e. by tuning the dipole moment’s direction of a peapod-like rigid rod. This is experimentally realised through the synthesis of monodisperse magnetoresponsive rods of desired diameter, length, and magnetic susceptibility [12].

Driven by the interest in the phase behavior of polar liquid crystals, earlier models of rod-like particles with a *single* longitudinal (or transversal) dipole moment have been intensively studied both by theory and numerical simulations [13–16]. We present here a numerical study of the self-assembly of a two dimensional system of stiff peapod-like straight filaments, composed of single magnetic dipolar beads that are rigidly linked one by one. A similar system was studied earlier by experiment [17] and simulations [18, 19]. The interaction is given by the superposition of the dipolar fields of each dipole bead. Differently of our previous work [19], in this study the direction of the dipole moment is altered with respect to the rod axis, opening the possibility of a plethora of new kinds of assembled clusters. Many experiments involving assemblies of colloids are actually done at surfaces and/or thin films [20–25], which motivates us to explore two dimensional (2*D*) systems.

We also study the connectivity properties of the present system by focusing on the percolation transition as a function of the direction of the dipole moment. The percolation transition is related to gelation in attractive-driven colloidal systems. Percolation behavior is also of great relevance in highly connected materials due to the possibility of enhancing the electrical and thermal conductivity [17, 26]. In addition, there is a relation with the change of the viscosity in systems with sufficient strong bond strength [27, 28].

The interaction strength may either increase or decrease the volume fraction required for percolation according to the definition of the separation at which two particles are considered to be connected, the dimensionality, and the proximity to the critical temperature [29]. As observed by Miller and Frenkel [30] for a system with adhesive hard spheres (AHS), the localization of the percolation threshold, the critical value of a given parameter where the percolation statistically happens, also depends on the interaction strength between particles, which is directly related with the temperature of the system. Therefore it is worth to explore possible routes under which the percolation transition is enhanced. Concerning the dimensionality, and, by using elongated particles, it was shown, in the 3*D* case, that an increase of the aspect ratio decreases the percolation threshold [18, 31], while the opposite behavior is observed in the 2*D* case [19]. In the present work we study the equilibrium configurations and the percolation transition as a function of the angular misalignment of the dipole moment with respect to the rod axis, and we show that larger values of this misalignment yields an enhancement of the percolation transition, i.e., an infinite extended cluster is formed for lower density.

The paper is organized as follows: our model system and simulation details are given in Sect. 2. The obtained different cluster configurations are presented in Sect. 3. The connectivity properties are discussed in Sect. 4. Our conclusions are given in Sect. 5.

## Model and simulation methods

**Fig 1:**
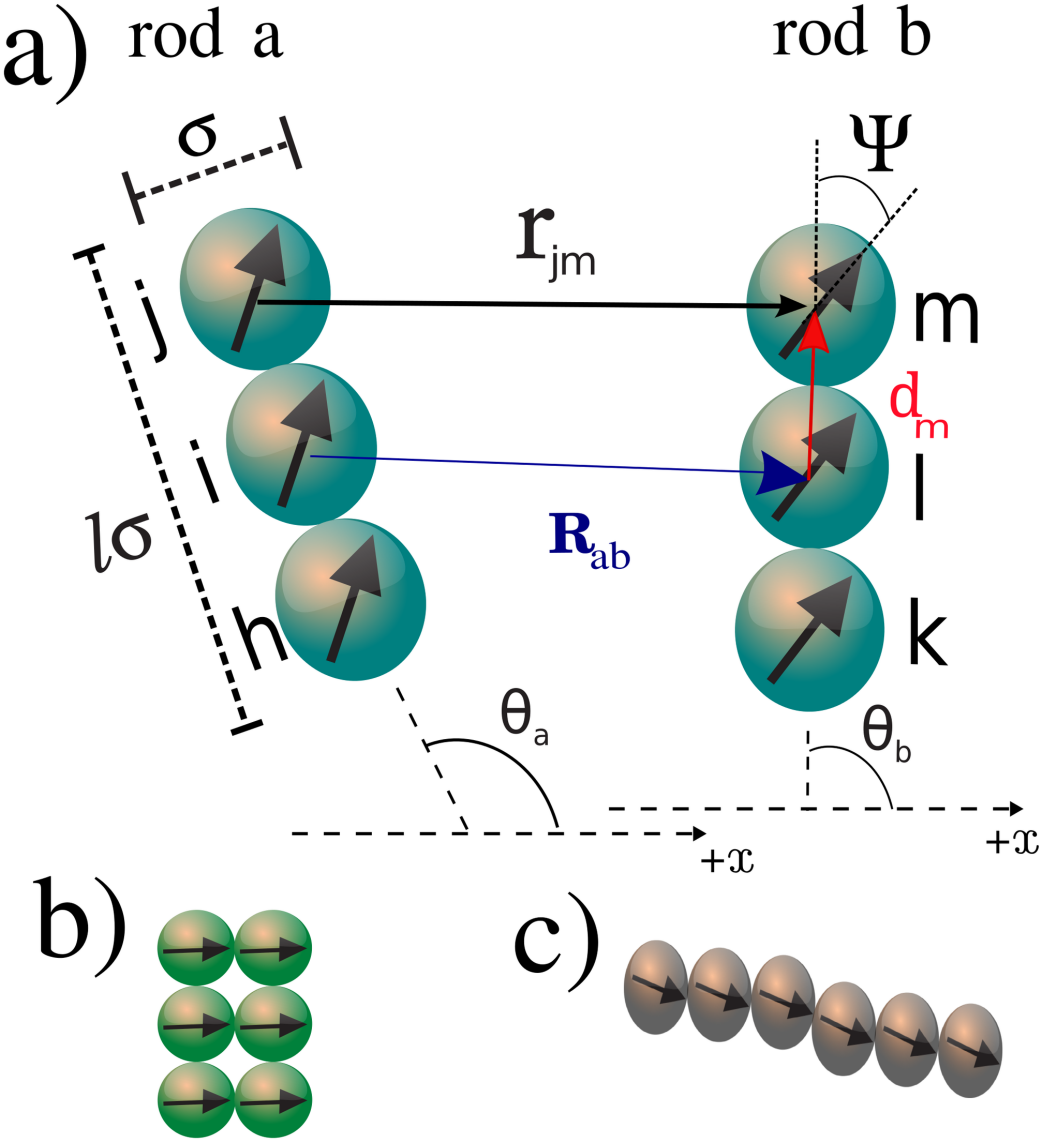
Schematic illustration of the interaction between two magnetic rods with: a) indication of the important parameters of the pair interaction potential; b) ribbon-like arrangement; c) head-to-tail arrangement.

Extensive Langevin Dynamics simulations were performed to study a two-dimensional (2*D*) system consisting of typically, unless stated otherwise, *N* = 840 identical stiff rods of aspect ratio *l* = 3. The phase behavior of a mono-dispersed system with the same aspect ratio was recently studied [32] and is considered an established reference system [33]. For suspensions studied experimentally, the aspect ratio *l* = 3 is in the lowest accessible limit [34]. The magnetic nature of the rod is simulated by attaching a point dipole of permanent magnetic moment *μ* at the center of each bead (see Fig 1). The orientation of the dipoles with respect to the axial direction of the rod is given by the angle Ψ, as illustrated in Fig 1. To model the dipolar particles we use a dipolar soft sphere (DSS) potential [19], consisting of the repulsive part of the Lennard-Jones (LJ) potential *u*^*rep*^ and a point-like dipole-dipole interaction part *u*^*D*^. The total interaction energy between rods *a* and *b* is the sum of the pair interaction terms between their respective dipolar spheres (DS):

$$\begin{array}{c} U_{a,b}\left(R_{a,b},\theta_{a},\theta_{b}\right)=\sum_{j\neq m}u_{j,m}, \end{array}$$ (1)

$$u_{j,m}=u^{rep}(r_{jm}^{a,b})+u^{D}(r_{jm}^{a,b},\mu_{j}^{a},\mu_{m}^{b}),$$ (2)

where:

$$u^{rep}=4\epsilon {\left(\frac{\sigma }{r_{jm}}\right)}^{12},$$ (3)

$$u^{D}=\frac{\mu_{j}\cdot \mu_{m}}{r_{jm}^{3}}-\frac{3(\mu_{j}\cdot r_{jm})(\mu_{m}\cdot r_{jm})}{r_{jm}^{5}},$$ (4)

with *σ* the diameter of each bead, and *ϵ* is the LJ soft-repulsion constant, **R**_*a,b*_ = **R**_*b*_ − **R**_*a*_ is the vector which connects the center of rod *b* with the center of rod *a*. The orientation of rods *a* and *b* are given by *θ*_*a*_ and *θ*_*b*_, respectively. The vector $r_{jm}^{a,b}$ connects the center of bead *m* of rod *b* with the center of bead *j* of rod *a* (see Fig 1). The force on bead *m* due to bead *j* is given by:

$$f_{jm}=-\nabla u_{jm}.$$ (5)

The torque on bead *m* is [19]:

$$\tau_{m}=\mu_{m}\times \sum_{m\neq j}B_{jm}+d_{m}\times \sum_{m\neq j}f_{jm},$$ (6)

where **d**_*m*_ is the vector connecting the center of bead *m* (rod *b*) with the center of rod *b* as illustrated in Fig 1, and **B**_*jm*_ is the magnetic field generated by the dipole moment *μ*_*j*_ at the position of the dipole *μ*_*m*_, which is given by:

$$B_{jm}=\frac{3(\mu_{m}\cdot r_{jm})r_{jm}}{r_{jm}^{5}}-\frac{\mu_{m}}{r_{jm}^{3}}.$$ (7)

The summations in Eq (6) are considered only for dipoles belonging to distinct rods. The orientation of the rods is given by the unitary vector **s** given by **s** = **d**_*m*_/ ∣ **d**_*m*_ ∣. The translational and rotational Langevin equations of motion of rod *b* with mass *M*_*b*_ and moment of inertia *I*_*b*_, are given by:

$$\begin{array}{c} M_{b}\frac{dv_{b}}{dt}=F_{b}-\Gamma_{T}v_{b}+\xi_{b}^{T}\left(t\right), \end{array}$$ (8)

$$\begin{array}{c} I_{b}\frac{d\omega_{b}}{dt}=N_{b}-\Gamma_{R}\omega_{b}+\xi_{b}^{R}\left(t\right), \end{array}$$ (9)

where **v**_*b*_ = *d* **R**_*b*_/*dt*, ***ω***_*b*_ is the angular velocity, **F**_*b*_ and **N**_*b*_ are the total force and torque acting on rod *b*, respectively, while Γ_*T*_ and Γ_*R*_ are the translational and rotational friction constants. $\xi_{b}^{T}$ and $\xi_{b}^{R}$ are the Gaussian random force and torque, respectively, which obey the following white noise conditions: $\langle \xi_{b}^{\alpha }\left(t\right)\rangle =0$, $\langle \xi_{b}^{\alpha }\left(t\right)\cdot \xi_{b^{\prime }}^{\alpha }\left(t^{\prime }\right)\rangle =2\Gamma_{\alpha }k_{B}T\delta_{bb^{\prime }}\delta \left(t-t^{\prime }\right)$, *α* = *T*, *R*. For rod-like particles, the translational friction constant is a combination of the parallel and perpendicular components with respect to the rod axis, so that the total translational diffusion coefficient is $D_{T}=\frac{1}{3}\left(D_{∥}+2D_{⊥}\right)$ for $D_{⊥}=\frac{1}{2}D_{∥}$ [35]. However, since the dynamical properties are not studied in the present work, for equilibrium simulations the values of Γ_*T*_ as well as Γ_*R*_ are irrelevant. We introduce the ratio Γ_*T*_ / Γ_*R*_ = 4/3 because such a ratio is already known to produce fast relaxation to equilibrium in similar systems [35].

We define the reduced unit of time as $t^{*}=t/\sqrt{\epsilon^{-1}M\sigma^{2}}$, where *M* is the mass of the rod. The energy is given in reduced units as U* = *U*/*ϵ*, the dipole moment in dimensionless units as $\mu^{*}=\mu /\sqrt{\epsilon \sigma^{3}}$, and the dimensionless distances as of r* = *r*/*σ*. Unless stated otherwise, the ratio of the thermal energy to the soft-sphere repulsion constant is chosen to be *k*_*B*_ *T*/*ϵ* = 0.1, where *ϵ*/*k*_*B*_ is the temperature unit and *k*_*B*_ is the Boltzmann constant. Periodic boundary conditions are taken in both spatial directions. Since the dipolar pair interaction falls off as (*r*^−3^), we take the simulation box sufficiently large such that no special long-range summation techniques [36] are needed. We define the packing fraction as *η* = *N*_*beads*_ *π*(*σ*/2)^2^/*L*^2^, where *N*_*beads*_ = 2520 is the total number of dipolar beads of the system and *L*^2^ is the simulation box area. Since *N*_*beads*_ = *lN*, we can rewrite the packing fraction as *η* = ρ*lπ/4, where ρ* is the dimensionless density ρ* = *ρσ*^2^, and *ρ* = *N*/*L*^2^. The reduced time step is typically in the range δt* = 10^−4^ − 10^−3^.

All simulations are performed at a very slow cooling rate. Initially, we set the temperature at *k*_*B*_ *T*/*ϵ* = 2 and slowly reduced it in steps Δ*k*_*B*_ *T*/*ϵ* = 0.05, each 5 × 10^5^ time steps, till the final temperature is reached. The quantities of interest are then averaged over more than 10^6^ time steps. All the beads from all rods have the same dipole moment whose magnitude we set as μ* = 1. Common experimental values of μ*^2^ at room temperature ranges in the interval 0.1 ≤ μ*^2^ ≤ 10. For example, in experiments [37] carried out using aqueous dispersions of superparamagnetic microspheres of ferrite grains (Estapor (R) from Merck—reference M1- 030/40) for *r* ≈ 205 *nm* and *M*_*s*_ ≈ 6 × 10^4^ *A*/*m*, the magnetization (*M*) of the particles is completely reversible and adjustable by an external magnetic field. If we consider *T* = 293 *K* and *M* ≈ 22, 6% of *M*_*s*_, we obtain μ* ≈ 1.

## Results and discussion

### Cluster formation

**Fig 2:**
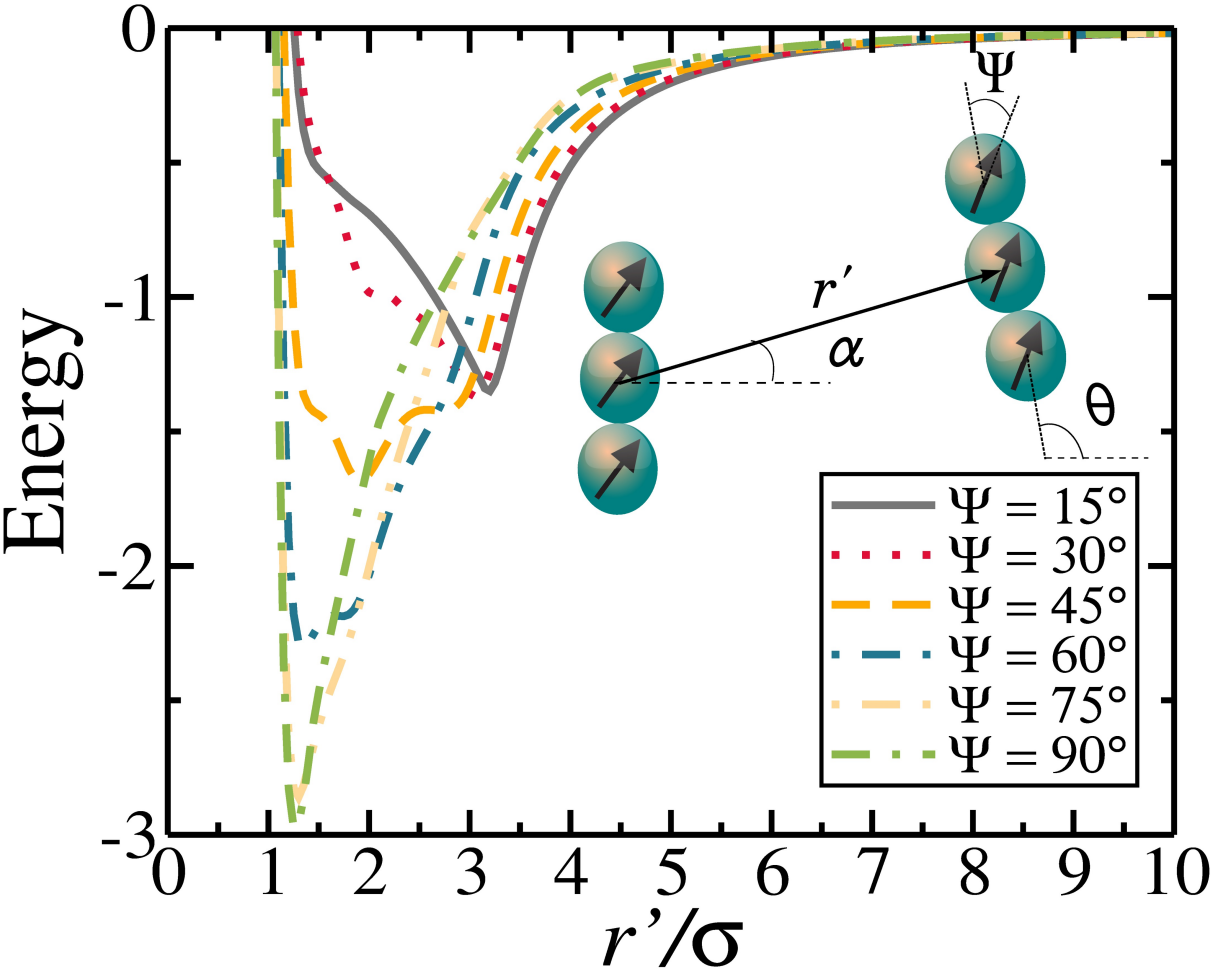
The pair interaction energy as a function of interrod separation (r′) minimized with respect to *α* and *θ*.

We first present the dependence of the DSS pair interaction potential on the separation between rods. The study of the pair interaction potential is needed in order to understand the nature of the resulting many-body interaction and to help us to set the values of the useful parameters that help to understand the self-assembled structures. The dependence of the DSS pair interaction potential as a function of the separation between rods and minimized with respect to some characteristic angles is presented in Fig 2. Rather different potential profiles are obtained with changing values of Ψ. For low values of Ψ (≤ 30°) the minima are located at *r*′/*σ* ≈ 3, which corresponds to the aspect ratio of the rods. The values of *α* and *θ* which minimize the pair-interaction energy indicate that rods are favourably in the head-to-tail bond. As Ψ is increased the position of the global minimum is displaced to smaller values of *r*′/*σ*, suggesting that the head-to-tail bond disappears, giving rise to the ribbon-like bond configuration (see Fig 1). Ribbon-like configurations are defined here as a side-by-side assembly as a consequence of the head-to-tail tendency of alignment between dipoles of beads in different rods and which are sufficiently displaced from the axial direction (Ψ > 45°). Similar ribbon-like configurations were very recently observed for microscopic magnetic ellipsoids [38, 39], and in peanut-shaped colloids [40]. For Ψ = 45° we have a mixing of head-to-tail and ribbon-like arrangements with intermediate bonding energy and separation.

In order to find the separation (*δ*_*c*_) used as a definition when two rods are bonded, we analyze the interrod separation related to the minimal energy value. From Fig 2, we observe that the largest interrod separation related to the global minimum is located around ≈ 3.4*σ* for Ψ = 15° and 1.4*σ* for Ψ = 90°. In the former the rods are in the head-to-tail arrangement, while in the latter they are in the ribbon-like configuration. In both cases, the shortest separation between beads of different rods is ≈ 1.4*σ* (bead-to-bead center distance). Therefore, we define here that two rods are bonded if the shortest separation between them is ≤ 1.4*σ*.

Since the attraction between magnetic rods becomes stronger for larger Ψ (Fig 2), we expect that the ribbon-like configurations will become more stable, implying that the formation of clusters is facilitated in the many-body case. To show that this is indeed the case, we analyze the degree of polymerization [41], defined as:

$$\Phi =\langle \frac{N_{c}}{N}\rangle ,$$ (10)

where *N*_*c*_ is the number of clustered rods and *N* is the total number of rods.

**Fig 3:**
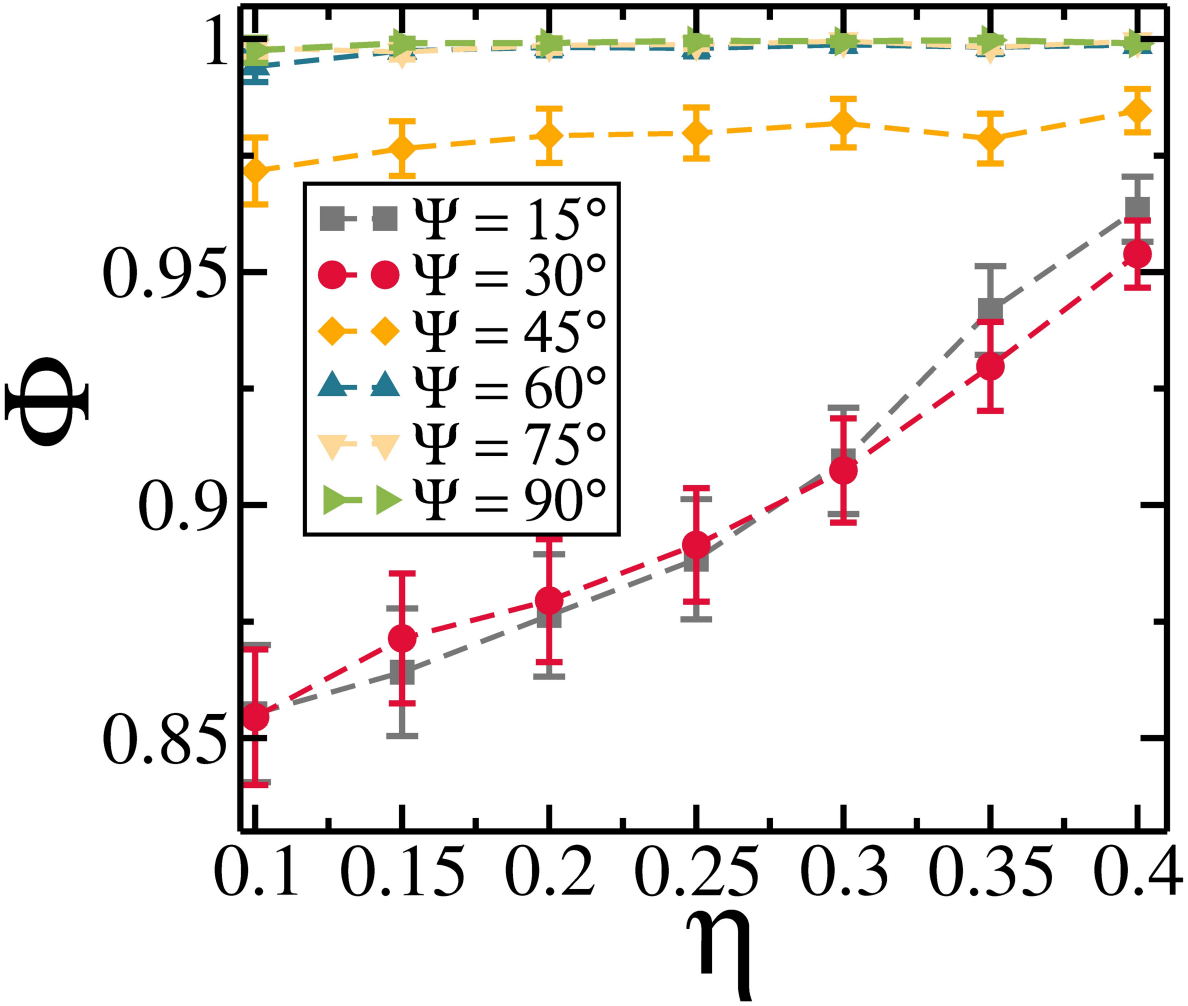
The polymerization as a function of the packing fraction *η* for different Ψ.

The polymerization as a function of the packing fraction *η* for different Ψ is presented in Fig 3. There is a clear distinction of *η*-dependence of the polymerization for distinct values of Ψ. For Ψ ≤ 30° the polymerization increases with increasing *η*, while for Ψ > 30°, the value of Ψ does not change as *η* is increased. In principle we would expect that the tendency for aggregation should be stronger as *η* is increased. However, such a behavior is found only when Ψ ≤ 30°, where the head-to-tail arrangement is mostly observed. For Ψ = 45°, the configurations are a mixture of head-to-tail and ribbon-like arrangements (minimum energy at *r*/*σ* ≈ 2, Fig 2) and the polymerization does not change significantly as *η* is changed. On the other hand, the larger Ψ, the larger is Φ, since, based on the minimized pair interaction function (Fig 2), there is an increase of interrod attraction specially when the rods form a ribbon-like arrangement. For Ψ ≥ 60°, the value of Φ does not change as Ψ is increased, and all the rods are connected to each other mostly arranged in the ribbon-like arrangement as the minimum energy is found for *r*/*σ* ≈ 1.4.

**Fig 4:**
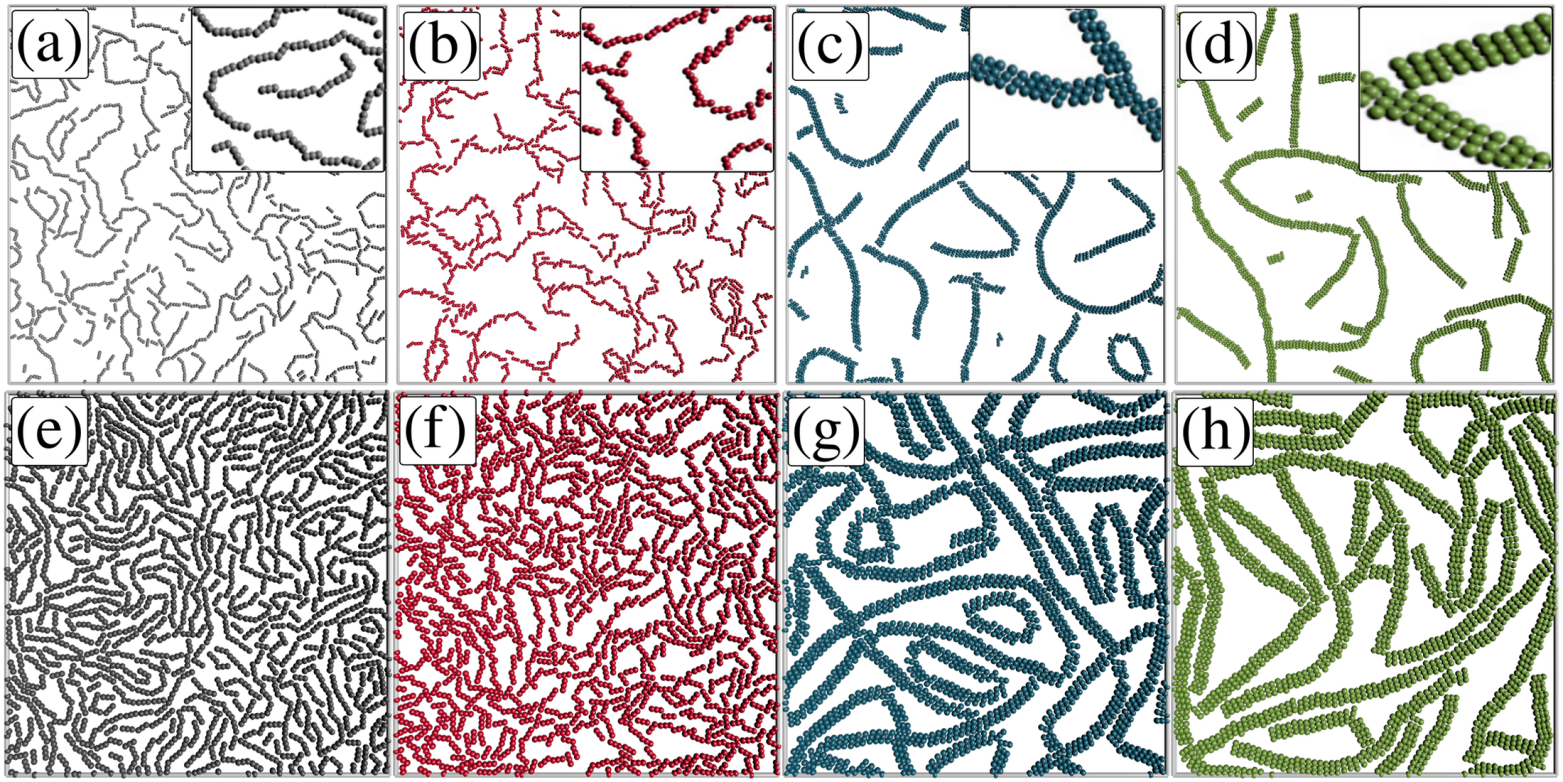
Some representative equilibrium configurations for *k*_*B*_ *T*/*ϵ* = 0.1 and packing fraction *η* = 0.1 with (a) Ψ = 15°; (b) Ψ = 30°; (c) Ψ = 60°; (d) Ψ = 90°; and for *η* = 0.3 with (e) Ψ = 15°; (f) Ψ = 30°; (g) Ψ = 60°; (h) Ψ = 90°. The insets are enlargments of part of the structures.

Some representative equilibrium configurations are presented in Fig 4 for packing fraction *η* = 0.1 and *η* = 0.3, and distinct Ψ. We observe that the head-to-tail arrangements are found for Ψ = 15° and Ψ = 30°, while the ribbon-like configurations are observed for Ψ = 60° and Ψ = 90°. Such arrangements of the rods can be better characterised by computing the pair correlation function [42]:

$$\begin{array}{c} g\left(r\right)=\frac{\langle \sum_{a}\sum_{b\neq a}^{N}\delta \left(r-R_{ab}\right)\rangle }{2N\pi r\rho^{*}}, \end{array}$$ (11)

where *R*_*ab*_ is the separation between the center of the rods *a* and *b* (see Fig 1).

**Fig 5:**
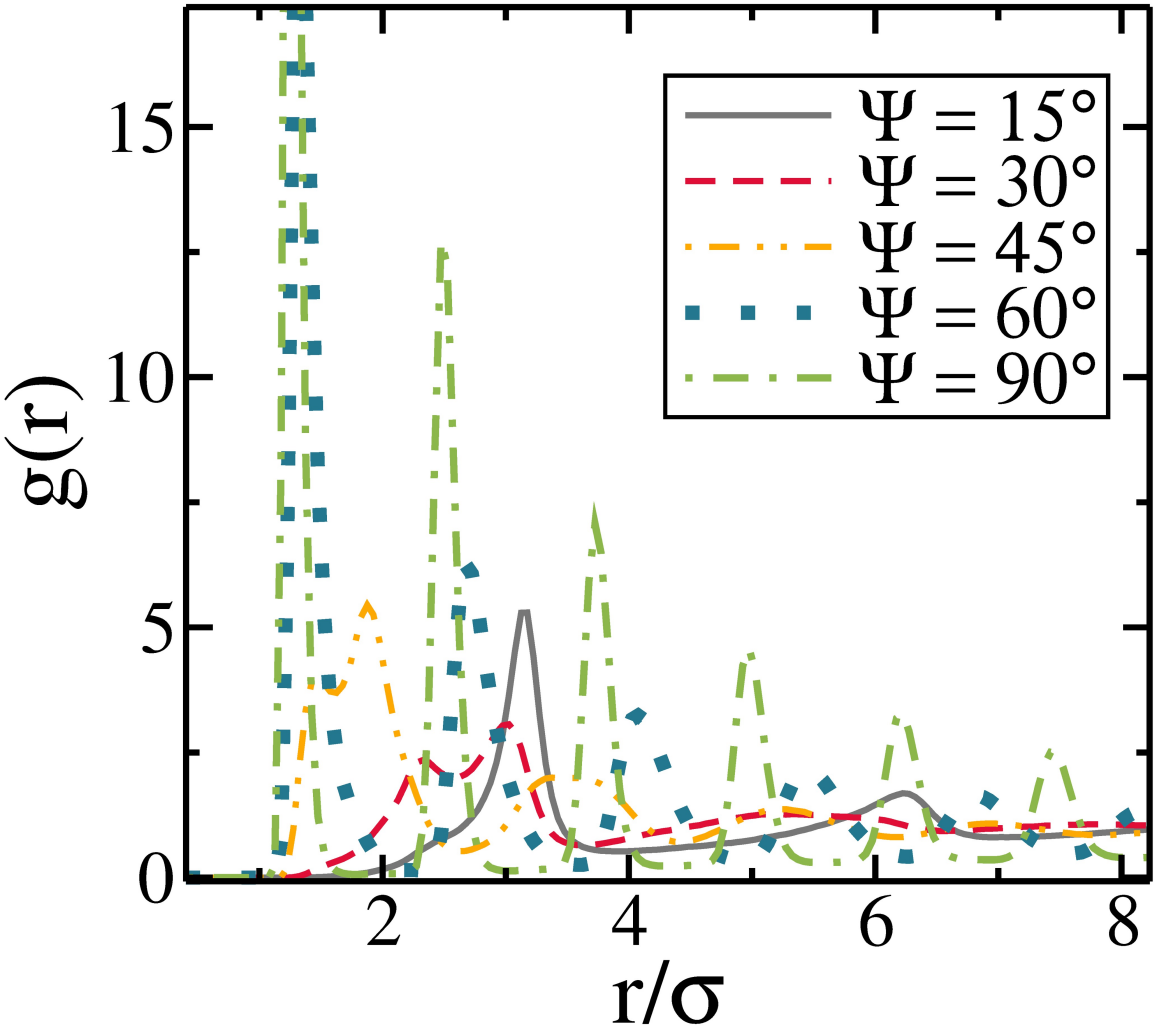
The pair correlation function for different values of Ψ with *η* = 0.1.

We present in Fig 5 the pair correlation function for *η* = 0.1 and for different values of Ψ. As shown, the position of the peaks changes towards smaller *r* for larger Ψ. For Ψ = 15° and Ψ = 30° the first largest peak is at *r*/*σ* ≈ 3, which coincides with the value of the aspect ratio of the rods and is associated with the head-to-tail alignment. When Ψ increases, multiple peaks proportional to *r*/*σ* ≤ 3 are found, and this is a consequence of the tendency to form side-by-side arrangements, e.g., for Ψ ≥ 60° the first largest peak is at *r*/*σ* ≈ 1.4. For intermediate values of the first largest peak position, i.e. *r*/*σ* ≈ 2 for Ψ = 45°, is a consequence of the mixed structures of head-to-tail and ribbon-like alignments, as expected by the pair interaction in Fig 2. For Ψ ≤ 45° the *g*(*r*) does not maintain a constant structure and it loses the long-range correlation, which is a typical behavior of liquid-like structures. On the other hand, for Ψ ≥ 60° the *g*(*r*) has regular peaks and long-range correlation, which is a typical behavior of solids. These results indicate, qualitatively, that the system goes from a liquid-like configuration (Ψ ≤ 45°) to a solid-like ribbon-like configuration (Ψ ≥ 60°), as a consequence of the fact that the attraction between rods in the ribbon-like configuration is much stronger than that observed in the linear head-to-tail arrangement, see Fig 2. In addition, such ribbon-like arrangements are expected to be more stable against thermal fluctuations.

### Connectivity properties

**Fig 6:**
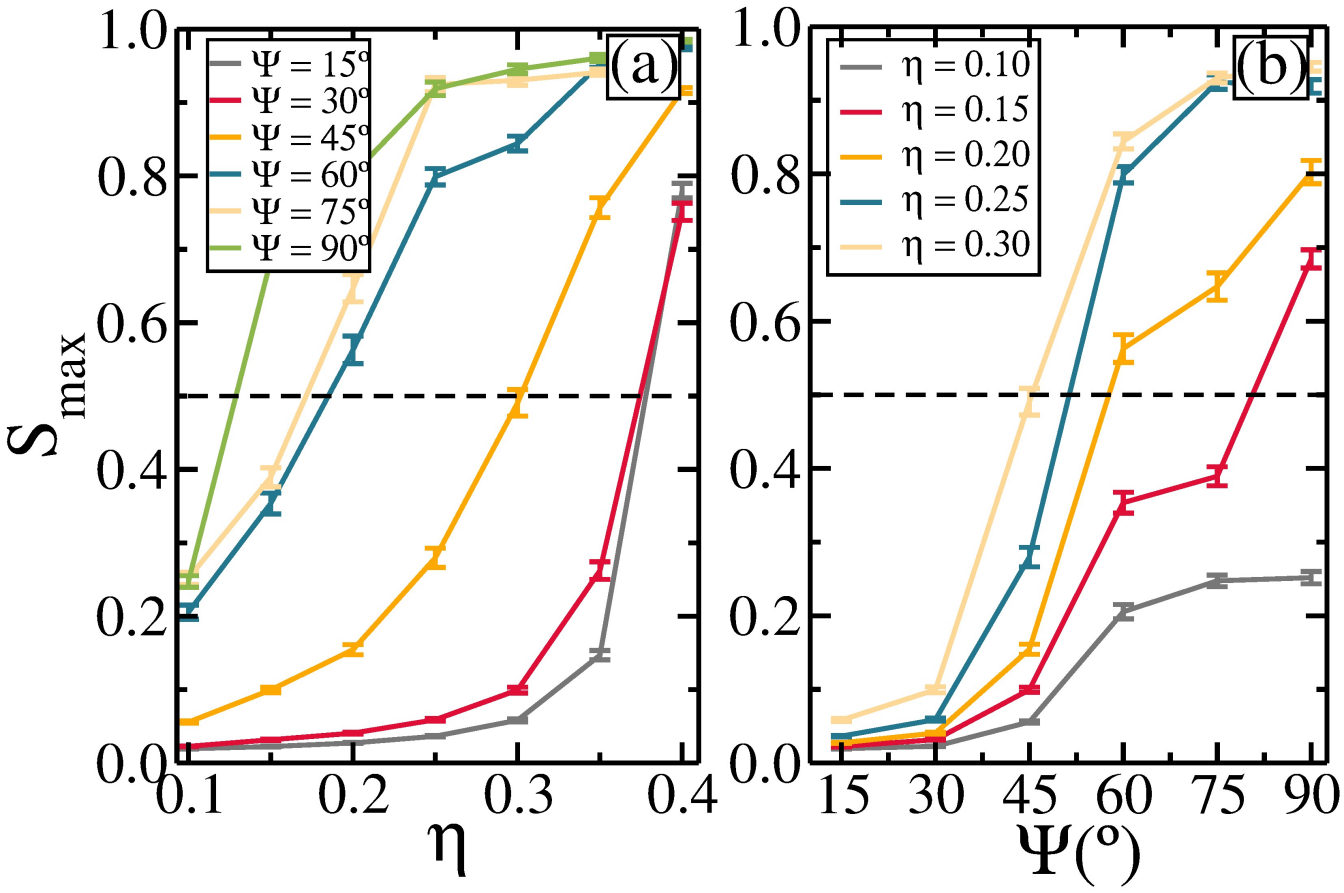
The average fraction of monomers in the largest cluster: (a) as a function of the packing fraction for different Ψ and (b) as a function of Ψ for different packing fractions. The horizontal dashed line at 0.5 refers to the percolation threshold.

Now we discuss the connectivity properties of the self-assembled structures. We focus our analysis on the study of the formation of percolated aggregates, which are defined as infinite connected clusters spanned over the system. The percolation transition is defined in the thermodynamic limit, where the average cluster size diverges [43]. We say that a configuration is percolated when, accounting for periodic boundary conditions, there is a percolating path [44], i.e., a cluster connected through opposite borders of the simulation box. A common feature of systems consisting of interacting particles subject to thermal fluctuations is that their bonds are transient. For low temperature, the lifetime of the bonds is sufficiently long, and the clusters are well defined over time. In our simulations, the temperature is one order of magnitude smaller than the average pair-interaction energy between rods, and the clusters are rather stable. In order to characterize the formation of the percolated clusters, we calculate an order parameter [45, 46] defined as the fraction of monomers in the largest clusters, *S*_*max*_, i.e.,

$$\begin{array}{c} S_{max}=\langle \frac{N_{larg}}{N}\rangle , \end{array}$$ (12)

where *N*_*larg*_ is the number of rods belonging to the largest cluster. Previous studies showed that for finite size systems, the percolation transition is well characterized when the size of the largest cluster is at least 50% of the total number of particles, i.e., *S*_*max*_ = 0.5 [45, 46]. Such an order parameter is useful to study the connectivity properties of the system, specially gelation [45, 47]. Here, we evaluate *S*_*max*_ as a function of the packing fraction and the orientation of the dipole moments with respect to the axial direction of the rod. *S*_*max*_ as a function of the packing fraction is presented in Fig 6(a) for different Ψ. The percolation transition is shifted towards smaller packing fraction with increasing Ψ, due to the stronger attraction between rods observed in these cases. As a consequence, the emergence of ribbon-like structures is facilitated. This is shown more clearly in Fig 6(b), where *S*_*max*_ is presented as a function of Ψ for different packing fractions. In all cases *S*_*max*_ increases with increasing Ψ, indicating that the rods interact more attractively, facilitating the formation of larger clusters. For small enough *η* (≲ 0.1) there is no formation of an infinite cluster independent of Ψ. Also, we observe that *S*_*max*_ saturates for Ψ ≳ 75°. The small *η* (≲ 0.1) and the tendency to form ribbons, which make the chains of the rods shorter in length, are the reasons for the absence of the extended clusters.

**Fig 7:**
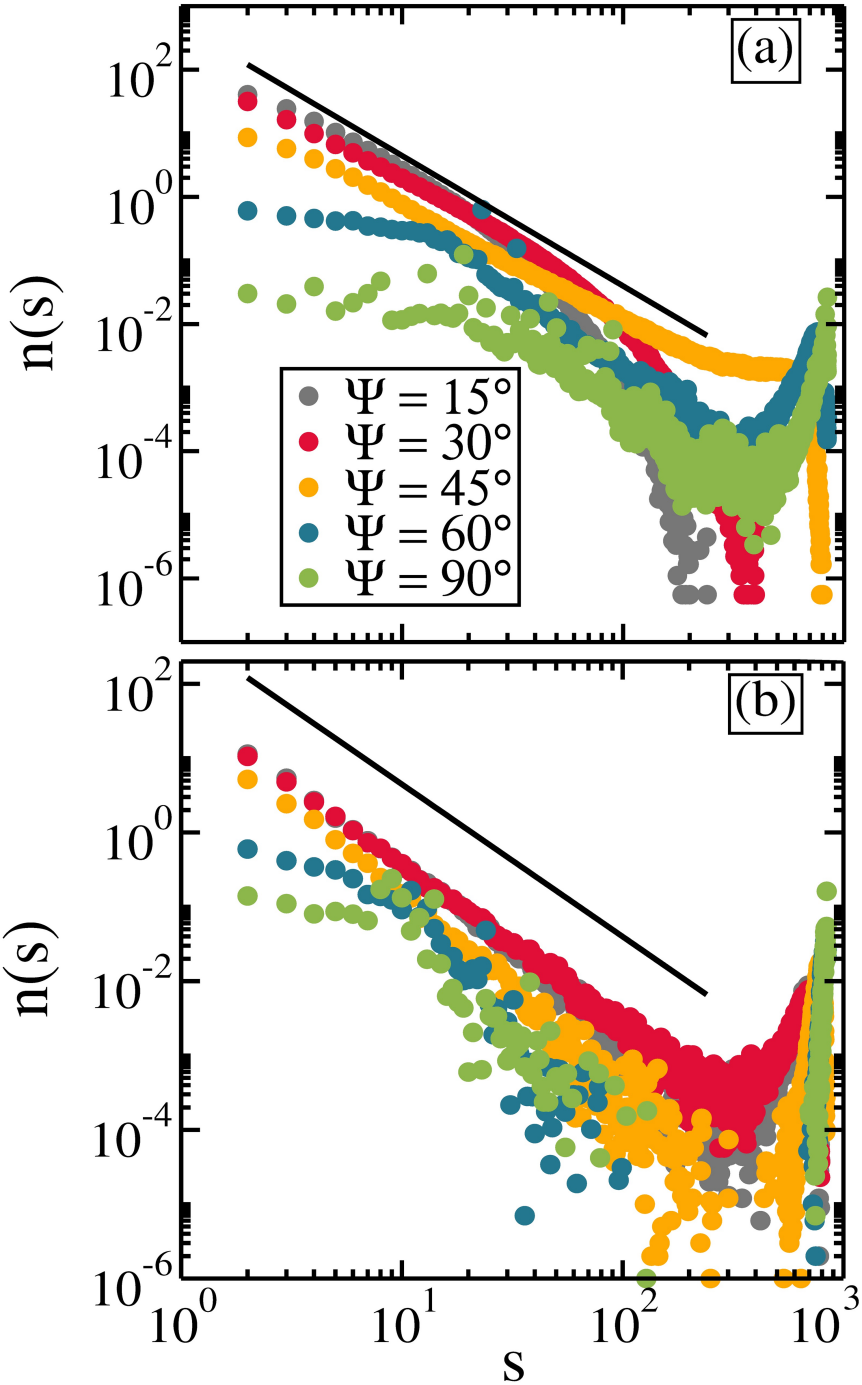
The cluster size distribution for different Ψ with (a) *η* = 0.2 and (b) *η* = 0.4. The solid line represents the function *n*(*s*) ∝ *s*^−2.05^. Axes are in log scale.

The connectivity properties can also be studied by the analysis of the cluster size distribution *n*(*s*), where *s* is the size of the cluster, i.e. the number of rods belonging to the cluster. In Fig 7 we present the average cluster size distribution for different Ψ with *η* = 0.2 [Fig 7(a)] and *η* = 0.4 [Fig 7(b)]. In general, *n*(*s*) decreases with increasing cluster size. The percolated configuration exhibits a single peak for large *s*, comparable to the system size, due to the finite size of the system considered in the simulations, and these states are denoted as random percolated [48, 49]. For *η* = 0.2, the system is percolated for 45° < Ψ ≤ 60° (see Fig 6(b)). Close to percolation (Ψ ≃ 45°) the *n*(*s*) curve presents a power law dependence, *n*(*s*) ∝ *s*^*τ*^, with exponent *τ* ≃ −2.05, which is related to the well-known 2*D* random percolation prediction [50](*τ* = −187/91 ≃ −2.05) valid in the thermodynamic limit. Similar random percolated structures were found in Langevin Dynamics of functionalized colloids [51]. For *η* = 0.4, a similar *s*-dependence for *n*(*s*), also with *τ* ≃ −2.05, is found for Ψ ≃ 30°, where the rods aggregate together through head-to-tail bonds, similar as shown in Fig 1(c). In both, *η* = 0.2 and *η* = 0.4, a similar power law dependence *n*(*s*)∼*s*^*τ*^ is observed, but with different mechanism. For *η* = 0.2, the rods aggregate to each other through head-to-tail bonds and the chains of rods start to merge into one another in a random way. For *η* = 0.4, the rods are connected to each other as ribbon-like arrangements forming long chains, which, in turn, are randomly bonded to each other. The *n*(*s*) curves also confirm that the percolation transition for the system with larger Ψ takes place for smaller *η*.

Another way to characterize the percolated structures is through the pair connectedness function *g*_*conn*_(*r*), defined as the conditional probability of finding a pair of particles separated by a distance *r*, both connected via a sequence of bonds, i.e., within the same cluster [52]. When an infinite cluster is present *g*_*conn*_(*r*) remains finite on every length scale. On the other hand, when a non-percolated structure is formed, we have *g*_*conn*_(*r*) → 0 for finite distances. A theory of the pair connectedness function has been previously developed for fluids as well as for lattice systems when the presence of physical clusters of particles in the system is explicitly taken into account [53]. In the limit when the whole system forms a single cluster, the pair connectedness function matches the pair correlation function [Eq (11)].

**Fig 8:**
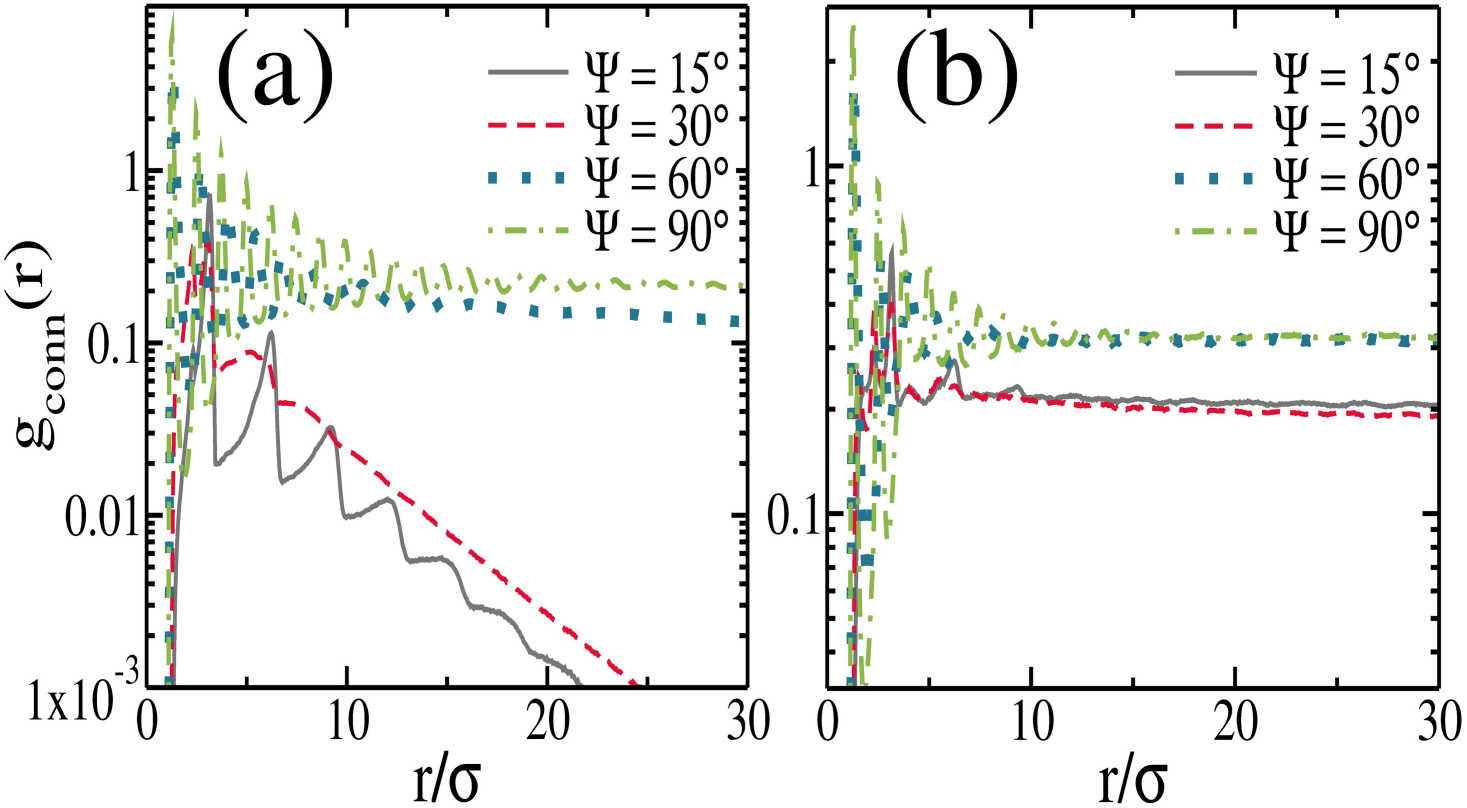
The pair connectedness function for different Ψ values and for packing fraction (a) *η* = 0.2 and (b) *η* = 0.4. The y-axes are in log scale.

In Fig 8(a) and 8(b) we show the pair connectedness function *g*_*conn*_(*r*) for packing fraction *η* = 0.2 and *η* = 0.4, respectively, and different values of Ψ. An infinite-size percolated cluster is observed when *g*_*conn*_ assumes non-zero values in the limit *r* → ∞, otherwise the cluster is not percolated. As can be observed the results are in agreement with those obtained through other distinct quantities, *n*(*s*) and *S*_*max*_.

**Fig 9:**
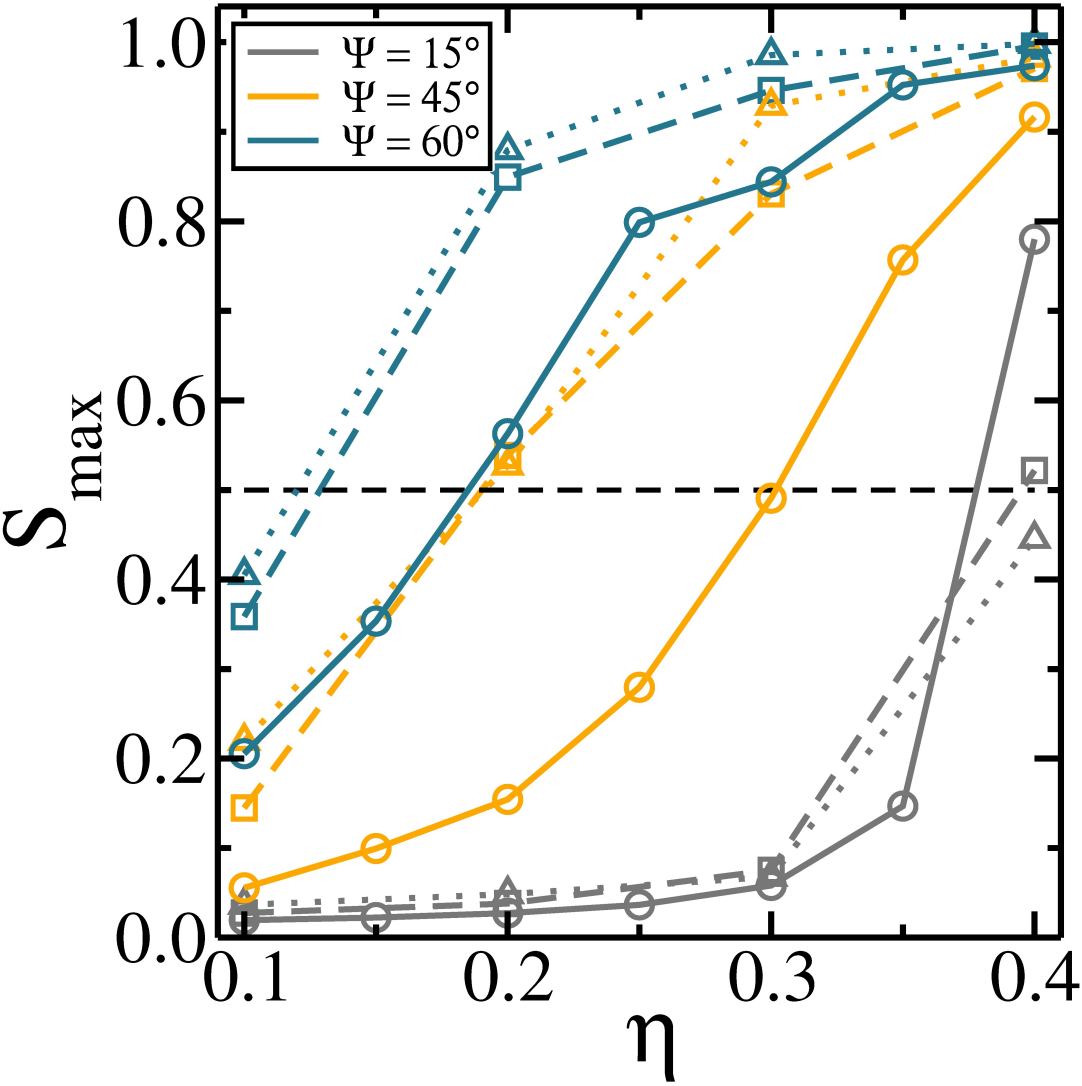
The average fraction of monomers in the largest cluster as a function of the packing fraction for different Ψ and different aspect ratios. Solid line with circle symbols: *l* = 3. Dashed line with square symbols: *l* = 5. Dotted lines with triangle symbols: *l* = 7.

In Fig 9 we briefly illustrate the effect of the increase of the aspect ratio on the percolation transition. For Ψ = 15°, larger values of *l* are associated with larger values of *S*_*max*_ at *η* = 0.4. Specifically, the system for *l* = 3 at *η* = 0.4 is clearly percolated, whereas for *l* = 5 it is in the transition region, and for *l* = 7 the system is not percolated. This shows that the percolation transition is suppressed for larger values of aspect ratio. Such a result agrees with our previous study [19]. For Ψ > 15°, the behavior is the opposite, i.e., the percolation transition is enhanced for larger values of *l*. This suggests that when the head-to-tail arrangements are dominant, the attraction between the rods is weaker, as already discussed, and the geometrical effects resultant from the increase of the rods aspect ratio (in a 2*D* system) make it harder to form large clusters [19]. This changes when the ribbon-like arrangement comes into play, because this arrangement has a stronger attraction and the geometrical effects resultant from the larger *η* and larger *l* no longer hamper the formation of large clusters, since the side-by-side arrangements is expected to be more present in the higher packing fraction regime.

### Effect of temperature

In this section we study the effect of temperature on the connectivity properties. The increase of temperature has a similar effect as to decrease the effective interparticle interaction. Although, from the perspective of reducing finite-size effects, it is advantageous to consider the percolation transition as a function of the density rather than as a function of temperature. However, it is worth to study the effect of temperature on the connectivity properties and to examine the stability of the clusters. It is expected that the percolation transition is hampered when temperature of an interacting system increases [52, 54], since stronger thermal fluctuations may break the transient bonds.

**Fig 10:**
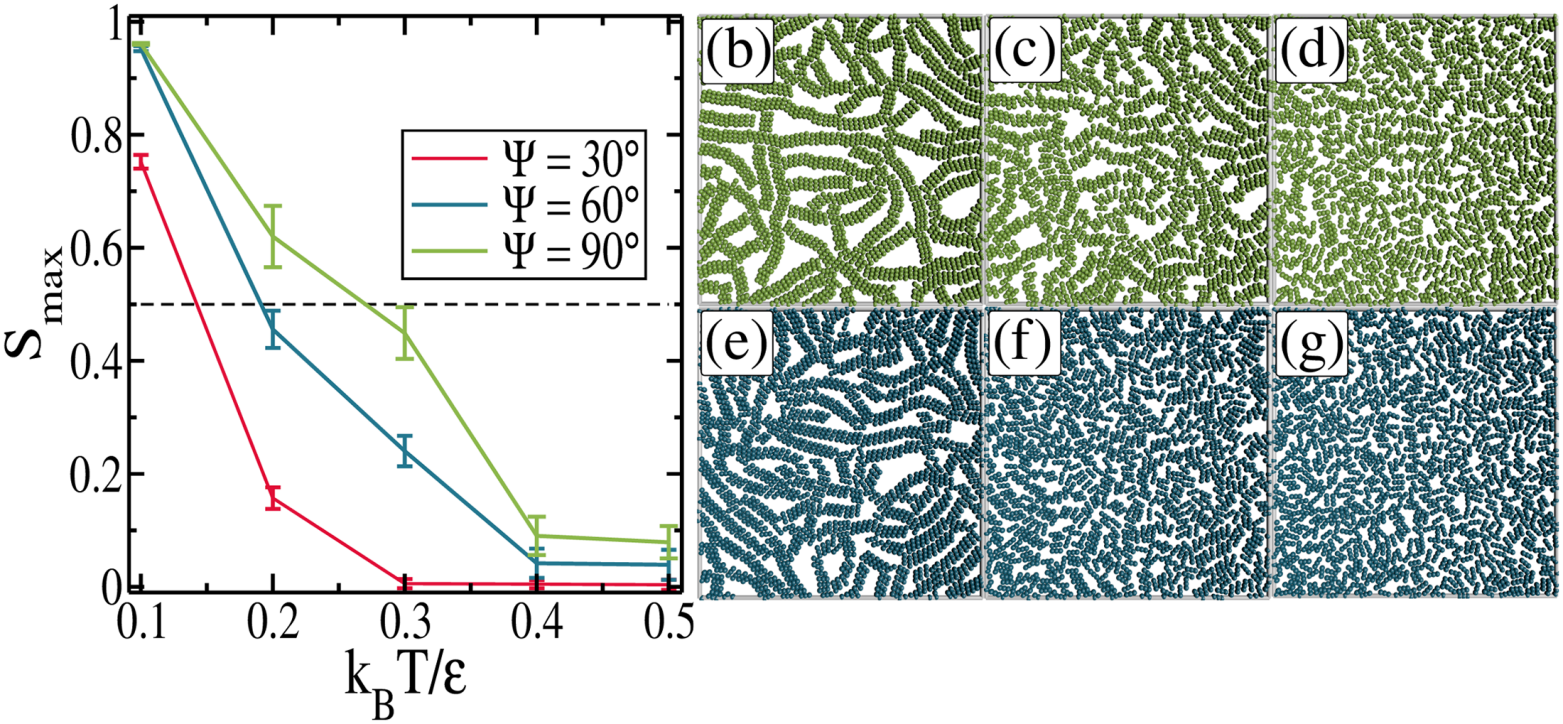
(a) The average fraction of monomers in the largest cluster as a function of temperature for *η* = 0.4. Some representative equilibrium configurations for *η* = 0.4 and: (b) Ψ = 90°, *k*_*B*_ *T*/*ϵ* = 0.1; (c) Ψ = 90°, *k*_*B*_ *T*/*ϵ* = 0.3; (d) Ψ = 90°, *k*_*B*_ *T*/*ϵ* = 0.5; (e) Ψ = 60°, *k*_*B*_ *T*/*ϵ* = 0.1; (f) Ψ = 60°, *k*_*B*_ *T*/*ϵ* = 0.3; (g) Ψ = 60°, *k*_*B*_ *T*/*ϵ* = 0.5.

In general, the ribbon-like arrangement is more stable against thermal fluctuations when compared to the head-to-tail structure. A specific example of the effect of temperature on the self-assembled configurations and on the percolation transition is presented in Fig 10(a), where the average size of the largest cluster (*S*_*max*_) is shown for *η* = 0.4 and for three values of Ψ. As pointed out previously, the system is percolated (*S*_*max*_ ≥ 0.5) for all the three considered values of Ψ for sufficiently small temperature (*k*_*B*_ *T*/*ϵ* = 0.1). In general, *S*_*max*_ decreases with increasing *k*_*B*_ *T*/*ϵ*, but how fast this happens depends on Ψ. The lower the value of Ψ, the lower the value of *S*_*max*_ for a given temperature. As a consequence, we may conclude that the percolated configurations of rods with larger Ψ are more stable against thermal fluctuations as compared to those with lower Ψ, and this is due to the formation of ribbon-like arrangements (see Fig 10). When compared to the head-to-tail bond between two rods, the ribbon-like one is stronger, since all dipoles of one rod are more strongly attracted to the dipoles of the other rod for Ψ > 45°, forming local head-to-tail bonds. We present in Fig 10(b)–10(g) snapshots of the ribbon-like structures, with (Ψ = 90° and Ψ = 60°). For temperatures *k*_*B*_ *T*/*ϵ* = 0.1, 0.3, and 0.5, highlighting how the clusters melt when submitted to higher temperatures. From these results, we may conclude that the percolation transition is enhanced for a system of peapod-like rods with larger values of Ψ even when it is analyzed as a function of temperature.

## Conclusion

In summary, we investigated a two-dimensional system consisting of magnetic rods using Langevin Dynamics simulations. Each rod is composed of 3 soft beads having a central point-like dipole which interact via a DSS potential. This model is motivated by recent experimental [17] and theoretical [18, 19] studies. A novelty of our system is the misalignment of the dipole moment of the individual beads with respect to the axial axis, opening the possibility of forming complex structures using nonspherical particles [12, 38–40]. Structural properties were investigated with particular attention to the dependence on the dipole’s direction Ψ and the packing fraction. We considered Ψ ranging from Ψ = 15° to Ψ = 90°.

Due to the dipole-dipole head-to-tail assembly tendency, the increase of Ψ produces a range of different configurations. As a consequence, we found that for Ψ ≥ 45° we observe ribbon-like structures which are energetically the most favorable. Given the preference of ribbon-like chain configurations we paid special attention to the appearance of a cluster extending over the whole system for a sufficient large packing fraction, i.e., the percolation configuration. We observed that the larger Ψ, the stronger the attraction between rods, facilitating the clustering process and favoring the formation of the infinite cluster. As a consequence, the larger Ψ, the smaller the packing fraction for the occurrence of the percolation transition. We found that by increasing the aspect ratio of the rods the packing fraction increases, and different geometrical effects are observed depending on the dominant kind of rod arrangement. For head-to-tail arrangements, the percolation transition is hampered with increasing *l*. The opposite behavior was observed when the ribbon-like arrangement becomes dominant.

We also investigated the temperature dependence of the percolated configuration by analysing how the average size of the cluster depends on *k*_*B*_ *T*/*ϵ*. We observed that percolated configurations with large Ψ are thermodynamically more stable as a consequence of the formation of ribbon-like bonds, which is characterized by local head-to-tail arrangements.

## References

[1] Demirörs AF, Stiefelhagen JC, Vissers T, Smallenburg F, Dijkstra M, Imhof A, et al Long-ranged oppositely charged interactions for designing new types of colloidal clusters. Physical Review X. 2015;5(2):021012.

[2] de Araújo J, Munarin F, Farias G, Peeters F, Ferreira W. Structure and reentrant percolation in an inverse patchy colloidal system. Physical Review E. 2017;95(6):062606 doi: 10.1103/PhysRevE.95.062606 28709279 10.1103/PhysRevE.95.062606

[3] Bianchi E, Blaak R, Likos CN. Patchy colloids: state of the art and perspectives. Physical Chemistry Chemical Physics. 2011;13(14):6397–6410. doi: 10.1039/c0cp02296a 21331432 10.1039/c0cp02296a

[4] Albrecht T, Bührer C, Fähnle M, Maier K, Platzek D, Reske J. First observation of ferromagnetism and ferromagnetic domains in a liquid metal. Applied Physics A. 1997;65(2):215–220. doi: 10.1007/s003390050569

[5] Gupta AK, Gupta M. Synthesis and surface engineering of iron oxide nanoparticles for biomedical applications. Biomaterials. 2005;26(18):3995–4021. doi: 10.1016/j.biomaterials.2004.10.012 15626447 10.1016/j.biomaterials.2004.10.012

[6] Duguet E, Mornet S, Vasseur S, Grasset F, Veverka P, Goglio G, et al Magnetic nanoparticle design for medical applications. Progress in Solid State Chemistry. 2006;34(2-4):237–47. doi: 10.1016/j.progsolidstchem.2005.11.010

[7] Hyeon T. Chemical synthesis of magnetic nanoparticles. Chem Commun. 2003;0:927–934. doi: 10.1039/b207789b 10.1039/b207789b 12744306

[8] Lemaire B, Davidson P. Ferr, eacute J., Jamet JP, Panine Dozov, I. I., Jolivet JP. Physical Review Letters. 2002;88:125507. 11909477 10.1103/PhysRevLett.88.125507

[9] Philip J, Shima P, Raj B. Enhancement of thermal conductivity in magnetite based nanofluid due to chainlike structures. Applied Physics Letters. 2007;91(20):203108 doi: 10.1063/1.2812699

[10] Abrikosov AI, Sacanna S, Philipse AP, Linse P. Self-assembly of spherical colloidal particles with off-centered magnetic dipoles. Soft Matter. 2013;9(37):8904–8913. doi: 10.1039/c3sm27128e

[11] Yener AB, Klapp SH. Self-assembly of three-dimensional ensembles of magnetic particles with laterally shifted dipoles. Soft Matter. 2016;12(7):2066–2075. doi: 10.1039/C5SM02648B 26768903 10.1039/c5sm02648b

[12] Singh H, Laibinis PE, Hatton TA. Rigid, superparamagnetic chains of permanently linked beads coated with magnetic nanoparticles. Synthesis and rotational dynamics under applied magnetic fields. Langmuir. 2005;21(24):11500–11509. doi: 10.1021/la0517843 16285833 10.1021/la0517843

[13] Zarragoicoechea G, Levesque D, Weis J. Monte Carlo study of dipolar ellipsoids. II. Search for an isotropic-nematic phase transition. Molecular Physics. 1992;75(5):989–998. doi: 10.1080/00268979200100771

[14] McGrother SC, Gil-Villegas A, Jackson G. The effect of dipolar interactions on the liquid crystalline phase transitions of hard spherocylinders with central longitudinal dipoles. Molecular Physics. 1998;95(3):657–673.

[15] Levesque D, Weis JJ, Zarragoicoechea GJ. Monte Carlo simulation study of mesophase formation in dipolar spherocylinders. Phys Rev E. 1993;47:496–505. doi: 10.1103/PhysRevE.47.496 10.1103/physreve.47.496 9960026

[16] Williamson DC, del Rio F. The isotropic–nematic phase transition in a fluid of dipolar hard spherocylinders. The Journal of chemical physics. 1997;107(22):9549–9558. doi: 10.1063/1.475252

[17] Birringer R, Wolf H, Lang C, Tschöpe A, Michels A. Magnetic nanorods: Genesis, self-organization and applications. Zeitschrift für Physikalische Chemie. 2008;222(2-3/2008):229–255. doi: 10.1524/zpch.2008.222.2-3.229

[18] Alvarez CE, Klapp SH. Percolation and orientational ordering in systems of magnetic nanorods. Soft Matter. 2012;8(28):7480–7489. doi: 10.1039/c2sm25636c

[19] Domingos JLC, Peeters FM, Ferreira WP. Self-assembly of rigid magnetic rods consisting of single dipolar beads in two dimensions. Physical Review E. 2017;96(1):012603 doi: 10.1103/PhysRevE.96.012603 29347093 10.1103/PhysRevE.96.012603

[20] Gao W, Rigout M, Owens H. Self-assembly of silica colloidal crystal thin films with tuneable structural colours over a wide visible spectrum. Applied Surface Science. 2016;380:12–15. doi: 10.1016/j.apsusc.2016.02.106

[21] Li L, Jiao X, Chen D, Li C. One-Step Asymmetric Growth of Continuous Metal–Organic Framework Thin Films on Two-Dimensional Colloidal Crystal Arrays: A Facile Approach toward Multifunctional Superstructures. Crystal Growth & Design. 2016;16(5):2700–2707. doi: 10.1021/acs.cgd.5b01817

[22] Shim TS, Estephan ZG, Qian Z, Prosser JH, Lee SY, Chenoweth DM, et al Shape changing thin films powered by DNA hybridization. Nature nanotechnology. 2017;12(1):41–47. doi: 10.1038/nnano.2016.192 27775726 10.1038/nnano.2016.192

[23] Yuan H, Zvonkina IJ, Al-Enizi AM, Elzatahry AA, Pyun J, Karim A. Facile Assembly of Aligned Magnetic Nanoparticle Chains in Polymer Nanocomposite Films by Magnetic Flow Coating. ACS Applied Materials & Interfaces. 2017;9(12):11290–11298. doi: 10.1021/acsami.7b02186 28240532 10.1021/acsami.7b02186

[24] Juàrez JJ, Bevan MA. Interactions and microstructures in electric field mediated colloidal assembly. The Journal of chemical physics. 2009;131(13):134704 doi: 10.1063/1.3241081 19814567 10.1063/1.3241081

[25] Velev OD, Gupta S. Materials Fabricated by Micro-and Nanoparticle Assembly–The Challenging Path from Science to Engineering. Advanced Materials. 2009;21(19):1897–1905. doi: 10.1002/adma.200801837

[26] Philip J, Shima P, Raj B. Nanofluid with tunable thermal properties. Applied physics letters. 2008;92(4):043108 doi: 10.1063/1.2838304

[27] Eberle AP, Wagner NJ, Castañeda-Priego R. Dynamical arrest transition in nanoparticle dispersions with short-range interactions. Physical review letters. 2011;106(10):105704 doi: 10.1103/PhysRevLett.106.105704 21469811 10.1103/PhysRevLett.106.105704

[28] Eberle AP, Castañeda-Priego R, Kim JM, Wagner NJ. Dynamical arrest, percolation, gelation, and glass formation in model nanoparticle dispersions with thermoreversible adhesive interactions. Langmuir. 2012;28(3):1866–1878. doi: 10.1021/la2035054 22148874 10.1021/la2035054

[29] Bug A, Safran S, Grest GS, Webman I. Do interactions raise or lower a percolation threshold? Physical review letters. 1985;55(18):1896 doi: 10.1103/PhysRevLett.55.1896 10031955 10.1103/PhysRevLett.55.1896

[30] Miller MA, Frenkel D. Phase diagram of the adhesive hard sphere fluid. The Journal of chemical physics. 2004;121(1):535–545. 15260575 10.1063/1.1758693

[31] Leung K, Chandler D. Theory of percolation in fluids of long molecules. Journal of Statistical Physics. 1991;63(5-6):837–856. doi: 10.1007/BF01029986

[32] May K, Eremin A, Stannarius R, Peroukidis SD, Klapp SH, Klein S. Colloidal Suspensions of Rodlike Nanocrystals and Magnetic Spheres under an External Magnetic Stimulus: Experiment and Molecular Dynamics Simulation. Langmuir. 2016;32(20):5085–5093. doi: 10.1021/acs.langmuir.6b00739 27119202 10.1021/acs.langmuir.6b00739

[33] De Miguel E, Rull LF, Chalam MK, Gubbins KE. Liquid crystal phase diagram of the Gay-Berne fluid. Molecular Physics. 1991;74(2):405–424. doi: 10.1080/00268979100102321

[34] May K, Stannarius R, Klein S, Eremin A. Electric-Field-Induced Phase Separation and Homogenization Dynamics in Colloidal Suspensions of Dichroic Rod-Shaped Pigment Particles. Langmuir. 2014;30(24):7070–7076. doi: 10.1021/la501120k 24866927 10.1021/la501120k

[35] Dhont JKG, Briels WJ. In: Rod-Like Brownian Particles in Shear Flow. Wiley-VCH Verlag GmbH & Co. KGaA; 2007 p. 147–216. Available from: http://dx.doi.org/10.1002/9783527617067.ch3a.

[36] Allen MP, Tildesley DJ. Computer simulation of liquids. Oxford university press; 1989.

[37] De Las Cuevas G, Faraudo J, Camacho J. Low-gradient magnetophoresis through field-induced reversible aggregation. The Journal of Physical Chemistry C. 2008;112(4):945–950. doi: 10.1021/jp0755286

[38] Martinez-Pedrero F, Cebers A, Tierno P. Orientational dynamics of colloidal ribbons self-assembled from microscopic magnetic ellipsoids. Soft matter. 2016;12(16):3688–3695. doi: 10.1039/C5SM02823J 26936015 10.1039/c5sm02823j

[39] Martinez-Pedrero F, Cebers A, Tierno P. Dipolar Rings of Microscopic Ellipsoids: Magnetic Manipulation and Cell Entrapment. Physical Review Applied. 2016;6(3):034002 doi: 10.1103/PhysRevApplied.6.034002

[40] Lee SH, Liddell CM. Anisotropic magnetic colloids: a strategy to form complex structures using nonspherical building blocks. Small. 2009;5(17):1957–1962. doi: 10.1002/smll.200900135 19415647 10.1002/smll.200900135

[41] Das SS, Andrews AP, Greer S. Living poly (*α*-methylstyrene) near the polymerization line. IV. Extent of polymerization as a function of temperature. The Journal of chemical physics. 1995;102(7):2951–2959. doi: 10.1063/1.468603

[42] Rapaport DC, Blumberg RL, McKay SR, Christian W, et al The art of molecular dynamics simulation. Computers in Physics. 1996;10(5):456–456.

[43] Miller MA, Blaak R, Lumb CN, Hansen JP. Dynamical arrest in low density dipolar colloidal gels. The Journal of chemical physics. 2009;130(11):114507 doi: 10.1063/1.3089620 19317545 10.1063/1.3089620

[44] Aharony A, Stauffer D. Introduction to percolation theory. Taylor & Francis; 2003.

[45] Liu Y, Pandey R. Sol–gel phase transitions in thermoreversible gels: Onset of gelation and melting. The Journal of chemical physics. 1996;105(2):825–836. doi: 10.1063/1.471891

[46] Chelakkot R, Gruhn T. Length dependence of crosslinker induced network formation of rods: a Monte Carlo study. Soft Matter. 2012;8(46):11746–11754. doi: 10.1039/c2sm07379j

[47] Bos MTA, van Opheusden JHJ. Brownian dynamics simulation of gelation and aging in interacting colloidal systems. Phys Rev E. 1996;53:5044–5050. doi: 10.1103/PhysRevE.53.5044 10.1103/physreve.53.5044 9964835

[48] Woodcock LV. Percolation transitions in the hard-sphere fluid. AIChE Journal. 2012;58(5):1610–1618. doi: 10.1002/aic.12666

[49] Godfrin PD, Valadez-Pérez NE, Castaneda-Priego R, Wagner NJ, Liu Y. Generalized phase behavior of cluster formation in colloidal dispersions with competing interactions. Soft matter. 2014;10(28):5061–5071. doi: 10.1039/C3SM53220H 24899107 10.1039/c3sm53220h

[50] Rubinstein M, Colby RH. Polymer physics. OUP Oxford; 2003.

[51] Dias CS and Braga C and Araújo NAM and da Gama MM Telo. Relaxation dynamics of functionalized colloids on attractive substrates. Soft matter. 2016; 5(12): 1550–1557. doi: 10.1039/C5SM02754C 10.1039/c5sm02754c 26661327

[52] Sciortino F, Tartaglia P, Zaccarelli E. One-dimensional cluster growth and branching gels in colloidal systems with short-range depletion attraction and screened electrostatic repulsion. The Journal of Physical Chemistry B. 2005;109(46):21942–21953. doi: 10.1021/jp052683g 16853852 10.1021/jp052683g

[53] Coniglio A, De Angelis U, Forlani A. Pair connectedness and cluster size. Journal of Physics A: Mathematical and General. 1977;10(7):1123 doi: 10.1088/0305-4470/10/7/011

[54] Schmidle H, Hall CK, Velev OD, Klapp SH. Phase diagram of two-dimensional systems of dipole-like colloids. Soft Matter. 2012;8(5):1521–1531. doi: 10.1039/C1SM06576A

