# Correction: Self-assembly and clustering of magnetic peapod-like rods with tunable directional interaction

**DOI:** 10.1371/journal.pone.0198007

**Published:** 2018-05-24

**Authors:** Jorge L. C. Domingos, François M. Peeters, W. P. Ferreira

There are errors in the Author Contributions. The correct contributions are: Conceptualization: JLCD, FMP, WPF. Data curation: JLCD. Formal analysis: JLCD. Funding acquisition: WPF, FMP. Investigation: JLCD, FMP, WPF. Methodology: JLCD, FMP, WPF. Project administration: WPF, FMP. Supervision: WPF, FMP. Writing–original draft: JLCD, FMP, WPF. Writing–review & editing: JLCD, FMP, WPF.
